# CD44 targets Wnt/β-catenin pathway to mediate the proliferation of K562 cells

**DOI:** 10.1186/1475-2867-13-117

**Published:** 2013-11-20

**Authors:** Guoqiang Chang, Hongju Zhang, Jian Wang, Yujuan Zhang, Hua Xu, Chijuan Wang, Hairui Zhang, Li Ma, Qinghua Li, Tianxiang Pang

**Affiliations:** 1 State Key Laboratory of Experimental Hematology, Institute of Hematology and Hospital of Blood Diseases, Chinese Academy of Medical Sciences and Peking Union Medical College, Nanjing Road 288, Tianjin 300020, China

**Keywords:** CD44, Wnt/β-catenin, Chronic myeloid leukemia, Proliferation, Cell cycle

## Abstract

**Background:**

Chronic myeloid leukemia is a clonal myeloproliferative disorder disease in which BCR/ABL plays an important role as an oncoprotein and molecular target. Despite the success of targeted therapy using tyrosine kinase inhibitors, CML remains largely incurable, most likely due to the treatment resistance after firstly chemical therapy. So know well the unique molecular pathway of CML is very important.

**Methods:**

The expressions of CD44 in different leukemia patients and cell lines were detected by real-time PCR and western blotting. The effects of CD44 on proliferation of K562 cells were determined using the MTT and colony formation assays, and even in a nude mouse transplantation model. Then, the cell cycle changes were detected by flow cytometric analysis and the early apoptosis of cells was detected by the annexin V/propidium iodide double-staining assay. The expressions of the cycles and apoptosis-related proteins p21, Cyclin D1 and Bcl-2 were analyzed by western blot and real-time PCR assay. Finally, the decreased nuclear accumulation of β-catenin was detected by western blotting and immunefluorescence.

**Results:**

Firstly, we showed that CD44 expression was increased in several kinds of leukemia patients and K562 cells. By contrast, the down-regulation of CD44 resulted in decreased proliferation with a G_0_/G_1_ arrest of cell cycle in K562 cells according to the MTT assay and the flow cytometric analysis. And no significant induction of both the early and late phases of apoptosis was shown by the annexin V-FITC and PI staining. During this process, p21 and cyclin D1 are the major causes for cell cycle arrest. In addition, we found CD44 down-regulation decreased the expression of β-catenin and increased the expression of phosphorylated β-catenin. The instability of Wnt/β-catenin pathway induced by increased expression of p-β-catenin resulted in a decreased nuclear accumulation in CD44 silenced K562 cells. In the nude mouse transplantation model, we also found the same results.

**Conclusions:**

These results show that K562 cells depend to a greater extent on CD44 for proliferation, and CD44 down-regulation may induce a cell cycle arrest through Wnt/β-catenin pathway. CD44 blockade may be beneficial in therapy of CML.

## Background

Chronic myeloid leukemia (CML), characterized by granulocytosis and splenomegaly, is a myeloproliferative disease and the disease course of CML is triphasic, starting with a chronic phase, progressing to an accelerated phase and ultimately ending in a terminal phase called blast crisis [1]. BCR/ABL is derived from chromosomal translocation (relocation of the portion of c-ABL gene from chromosome 9 to the portion of BCR gene locus on chromosome 22 t (9;22)), yielding the Philadelphia (Ph) chromosome that is present in over 90% of CML [2,3]. The Philadelphia chromosome in CML gives rise to constitutively active protein tyrosine kinase product BCR-ABL, which is important because in patients with CML, there is clonal expansion of hematopoietic cells that express this fusion gene. Moreover, continued expression of BCR-ABL is required for sustained proliferation of leukemic cells in mouse models of CML [4,5]. Inhibition of BCR-ABL with kinase inhibitors such as imatinib mesylate in the treatment of Ph^+^ CML is the current standard therapy, but it is highly effective in controlling but not curing the disease. This is largely due to the inability of these kinase inhibitors to kill leukemia stem cells (LSCs) responsible for disease relapse [2]. This ‘native’ resistance of LSCs in CML to imatinib and other kinase inhibitors suggests that the kinase somehow turns on unique molecular pathways in LSCs through both kinase-dependent and, more importantly, kinase-independent mechanisms [6].

To resolve the matter related to the drug resistance of LSCs in CML, it is essential to fully understand the molecular mechanisms in both kinase-dependent and kinase-independent pathways in CML. It is particularly crucial to identify the key genes that have significant roles in their survival and self-renewal. Emerging studies show that CD44 is an important biomarker of a cellular subpopulation (cancer stem cells, CSCs), which are capable of self-renewal and have the capacity for initiation, progression, invasion, metastasis, tumor recurrence, and resistance to chemo- and radiotherapy [7]. CD44 denotes a large family of transmembrane glycoproteins that are expressed in a variety of cells and tissues and plays a critical role in a variety of cellular behaviors, including adhesion, migration, invasion, and survival [8]. Daniela *et al.* also found CD44 was indispensable for BCR-ABL-expressing leukemic stem cell to initiate CML and CD44 blockade decreased engraftment and impaired induction of CML-like myeloproliferative disease [9].

The other key signal is Wnt/β-catenin, which are secreted signaling molecules that influence both development and cancer. Wnt/β-catenin regulates the differentiation of limbs, brain, kidney, and the reproductive tract in mice [10,11]. In addition to its importance in normal development, dysregulation of the Wnt/β-catenin pathway has potent oncogenic effects. Mutations in APC as well as β-catenin, a key mediator of Wnt/β-catenin signaling, are also found in a majority of sporadic colon cancers, hepatocellular carcinoma, as well as thyroid cancer, and ovarian cancer [12]. The fact is that Wnt/β-catenin signaling is dysregulated in multiple solid cancers together with its observed influence on hematopoietic stem and progenitor cells [13-16]. In blastcrisis CML patients, β-catenin is activated in myeloid progenitors and the activated β-catenin translocates to the nucleus [4], where it interacts with lymphoid enhancer/T-cell transcription factors and regulates the expression of genes. Also, β-catenin has been shown to be involved in BCR-ABL leukemogenesis. BCR-ABL stabilizes β-catenin in myeloid cells through induction of tyrosine phosphorylation and activation of β-catenin in BCR-ABL-positive granulocyte-macrophage progenitors from blastic phase CML patients facilitates the acquisition by these cells of properties of LSCs [1].

In this study, we used K562 chronic myeloid leukemia cells *in vitro* and *in vivo* to provide further evidence that CD44 and its target- β-catenin are essential for survival and self-renewal of CML cells.

## Results

### Expression of CD44 in leukemia

**Figure 1:**
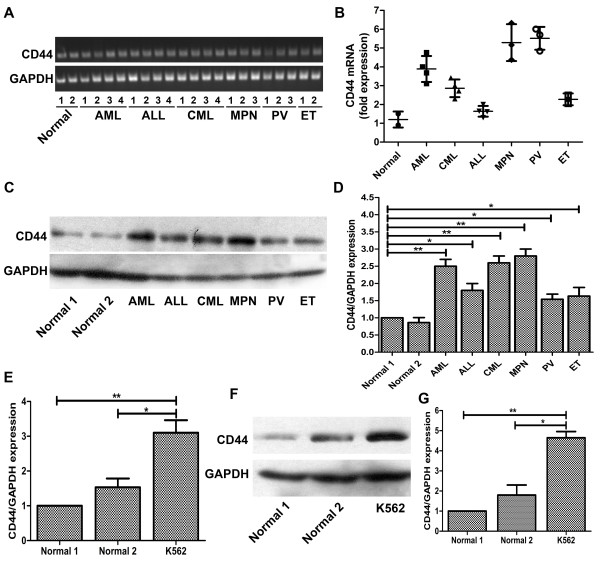
**Expression of CD44 in patients’ samples and leukemic cells. (A/B)** Expression patterns of CD44 in leukemic patients’ samples were analyzed by PCR and real-time quantitative PCR analysis. PCR reactions were performed on templates of cDNA from different patients’ cells using a set of primers as in Materials and methods. **(C)** The protein expressions of CD44 in different patients were analyzed by western blotting. **(D)** The relative protein expression of CD44 compared to GAPDH. **(E)** Real-time quantitative PCR analysis of CD44 gene expression in normal volunteers and K562 cells. **(F)** The protein expressions of CD44 in normal controls and K562 cells. **(G)** The relative protein expression of CD44 compared to GAPDH. For real-time quantitative PCR and western blotting, GAPDH was used as an internal control. *, P < 0.05, **, P < 0.01, compared with the control.

We first compared CD44 expression of patients with different leukemias by PCR and real-time PCR. We chose 4 acute myeloid leukaemia (AML) patients, 4 chronic myeloid leukemia (CML) patients, 4 acute lymphoblastic leukemia (ALL) patients, 3 myeloproliferative neoplasm (MPN) patients, 3 polycythemia vera (PV) patients, 2 essential thrombocythemia (ET) patients and 2 healthy volunteers to evaluate the expressions of CD44. The real-time PCR analysis revealed that the expressions of CD44 in leukemia patients were higher than that in healthy subjects, although the degrees of discrepancy were different (Figure 1A and B). Then we respectively chose one patient from each kind of diseases to compare the protein expression of CD44 between leukemia patients and healthy volunteers. Although the protein results of western blotting were little different from the RT-PCR results, all protein expressions of CD44 in patients were higher than that in normal controls (Figure 1C and D). And we also detected the expression of CD44 in K562 chronic myeloid leukemia cells. Compared with CD44 expression in two healthy volunteers, CD44 level in K562 cells was higher by nearly 3 fold (Figure 1E). Results of western blotting also showed the distinctive signals for the bands of CD44 in these samples. The results showed the protein expression of CD44 in K562 cells was higher, similar to our previous findings in mRNA (Figure 1F and G). These data indicate that the expression of CD44 in leukemia patients is higher than that in healthy volunteers.

### CD44 down-regulation inhibits the proliferation of K562 cells

**Figure 2:**
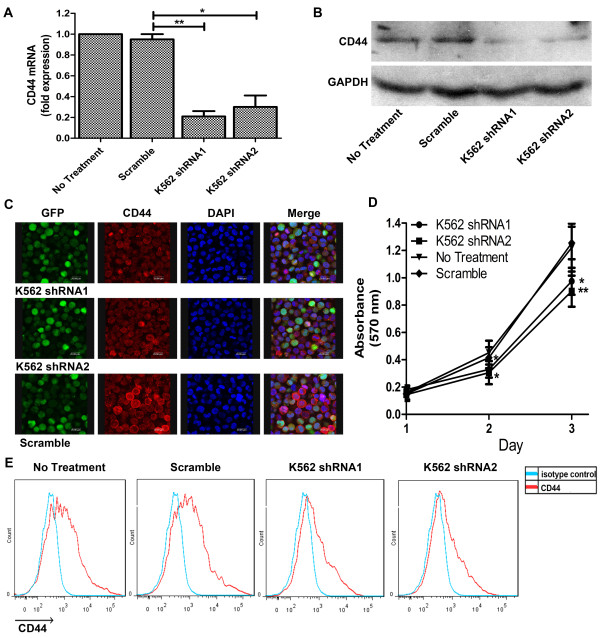
**Decreased proliferation in K562 cells upon CD44 silencing. (A/B)** Down-regulation of CD44 mRNA/protein expression in K562 cells after CD44 interference. **(C)** Confocal laser microscope showing localization of CD44 in K562 cells after transfection. The green panel is GFP; the red panel is CD44 and the blue panel is cell nuclear. **(D)** Effect of CD44 shRNA on the proliferation of K562 cells as assessed by MTT assay. **(E)** CD44 surface expression detected by flow cytometry. CD44 (red), isotype control (green). Results are shown as mean ± SD of three independent experiments, each experiment in triplicate. *, P < 0.05, **, P < 0.01, compared with the control.

According to the above results, the level of CD44 expression may be associated with aggressive tumor cells behaviors. So, to confirm the function of CD44 on proliferation, three CD44 shRNA plasmids were constructed and transfected into K562 cells, which express a higher level of CD44 and exhibit a more proliferative phenotype. After transfection, CD44 expression in K562 cells markedly decreased compared with control. In addition, the shRNA1 and 2 plasmids were more effective in decreasing the expression of CD44 (Figure 2A and B). As a membrane-type protein, the expression of CD44 on the membranes of K562 cells after transfection was detected by confocal laser microscope. The results showed that the membrane-type CD44 expression was also significantly decreased as the results of western blotting, and the ring forms of CD44 expression around the membrane of K562 cells were broken and some of them even disappeared (Figure 2C). We also tested the membrane-type CD44 expression by using flow cytometry and the results showed that the membrane-type CD44 expression was down-regualted compared to the control (Figure 2E). Down-regulation of CD44 in K562 cells resulted in a dramatic change of proliferation. We performed the nonradioactive quantification of cell proliferation and cell viability (3-[4,5-dimethylthiazol-2-yl]-2,5- diphenyl tetrazolium bromide (MTT) assay) for investigating the proliferation state of K562 cells. The result of MTT assay showed that when the expression of CD44 in K562 was down-regulated, the proliferation of K562 cells was significantly decreased. This decreased proliferation of these myeloid cells also correlated with decreased CD44 expression (Figure 2D).

### CD44 down-regulation induces a G_0_/G_1_ arrest in K562 cells

**Figure 3:**
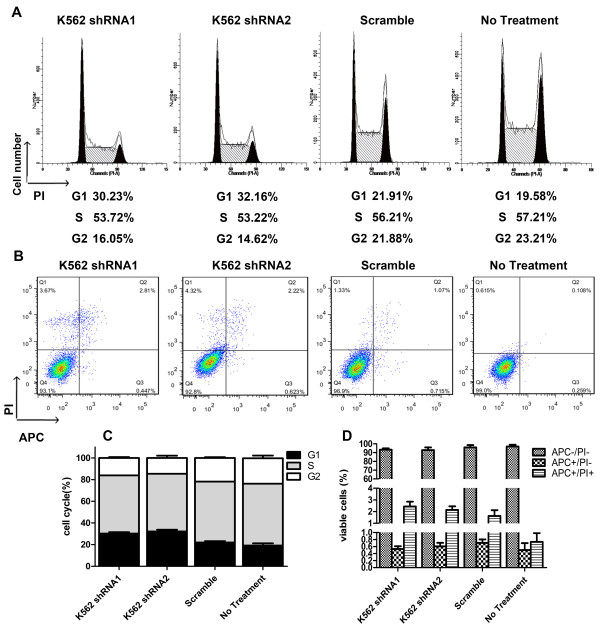
**CD44 down-regulation arrests K562 cells in the G**_**1 **_**phase of cell cycle. (A)** Cell cycle distribution of K562 cells before and after transfecting with CD44 shRNA. At the figures, cells were washed with PBS, and stained for DNA with propidium iodide as described in Materials and methods. Cell cycle distribution was then determined by FACS analysis. **(B)** Cell death distribution of K562 cells after treatment with CD44 shRNA. At the figures, cells were washed with PBS, and stained with APC-Annexin V and propidium iodide as described in Materials and methods. Cell death distribution was then determined by FACS analysis. **(C)** The relative distribution of cell cycle of K562 cells after transfection. **(D)** The relative distribution of cell death of K562 cells after transfection.

Cell cycle arrest is a common feature of cells undergoing terminal differentiation and defective proliferation. Based on the growth inhibitory effects of CD44 down-regulation on myeloid leukemia cell lines, we investigated their cell cycle progression of CD44 silencing K562 cells. The DNA contents of K562 cells after transfection were analyzed by FACS. Surprisingly, we observed a change in cell cycle of K562 cells when CD44 expression was inhibited compared to both K562 cells and cells treated with scramble plasmids. The results showed that CD44 knockdown K562 cells underwent a G_0_/G_1_ arrest. The proportion of cells in G_0_/G_1_ phase increased from 21% (controls) to 32% in CD44 silencing K562 cells (Figure 3A). This was mirrored by a decrease in the proportion of cells in the S and G_2_ phase from 56% (controls) to 53% in CD44 silencing K562 cells and from 21% (controls) to 14% in CD44 silencing K562 cells respectively (Figure 3A and C).

To investigate if the reduced cell proliferation was due to cell death, the levels of apoptosis were measured in K562 cells with reduced CD44 levels by flow cytometry. The percentage of APC-Annexin V single positive cells was not significantly different compared to control cells, indicating that the reduced cell proliferation was not due to cell death (Figure 3B and D). These data suggest that the growth inhibitory effect of CD44 suppression on K562 chronic myeloid cells is, in part, due to its effect on cell cycle arrest progression.

### CD44 down-regulation induces the expression of p21 and down-regulates the expression of cyclin D1

**Figure 4:**
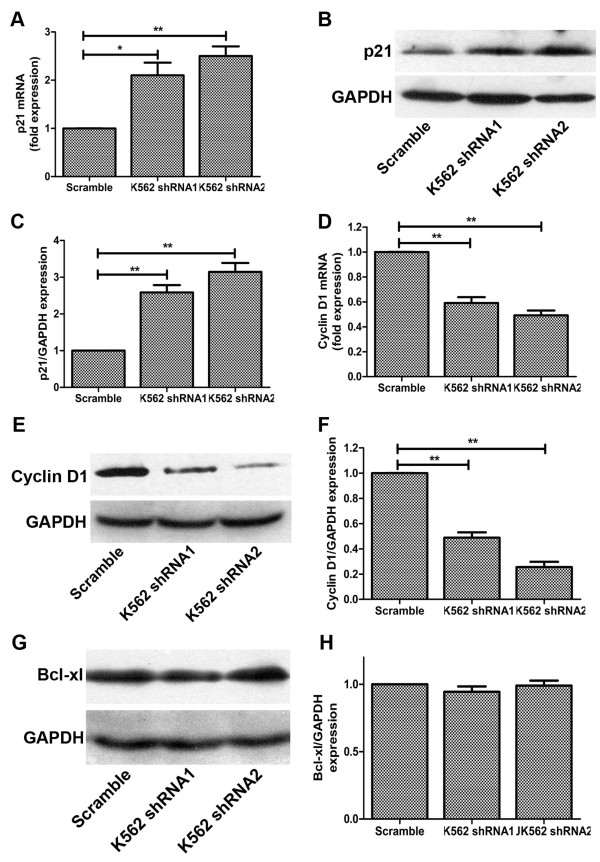
**CD44 down-regulation induces the expression of p21 and down-regulates the expression of major cell cycle regulatory protein-cyclin D1. (A/B)** Increased p21 mRNA/protein expression in CD44 silenced K562 cells. **(C)** The relative protein expression of p21 compared to GAPDH. **(D/E)** Decreased cyclin D1 mRNA/protein expression in K562 cells after CD44 interference. **(F)** The relative protein expression of cyclin D1 compared to GAPDH. **(G/H)** Unchanged protein expression of Bcl-xl. For real-time quantitative PCR and western blotting, GAPDH was used as an internal control. *, P < 0.05, **, P < 0.01, compared with the control.

Based on the effects of CD44 on G_1_ phase accumulation, we hypothesized the role of major G1 regulatory proteins. We examined the effect of CD44 shRNA on p21 protein expression. Our results show that CD44 shRNA treatment to K562 cells caused marked up-regulation of p21 protein expression (Figure 4A-C). Interestingly, increased p21 stability could account for the defects in proliferation and G_1_ arrest. P21 was discovered as a component of a quaternary complex consisting of cyclin D1, a CDK, the proliferating cell nuclear antigen (PCNA), and p21. So we also detected the cyclin D1 expression in CD44 silencing K562 cells. The results of western blotting showed that the protein expression of cyclin D1 was significantly decreased by CD44 down-regulation (Figure 4D-F), and this inhibition of cyclin D1 was correlated with increased level of p21. We also investigated the expression of anti-apoptotic protein Bcl-xl to verify the results of cells death assay. And the results of western blotting showed that the expression of Bcl-xl was not significantly changed (Figure 4G-H). These data suggest that induction of G_0_/G_1_ arrest by CD44 down-regulation in K562 cells may be mediated by increase in p21 levels and decrease in cyclin D1 levels.

### The instability of β-catenin caused by increase of p-β-catenin expression contributes to CD44-mediated inhibition of proliferation

**Figure 5:**
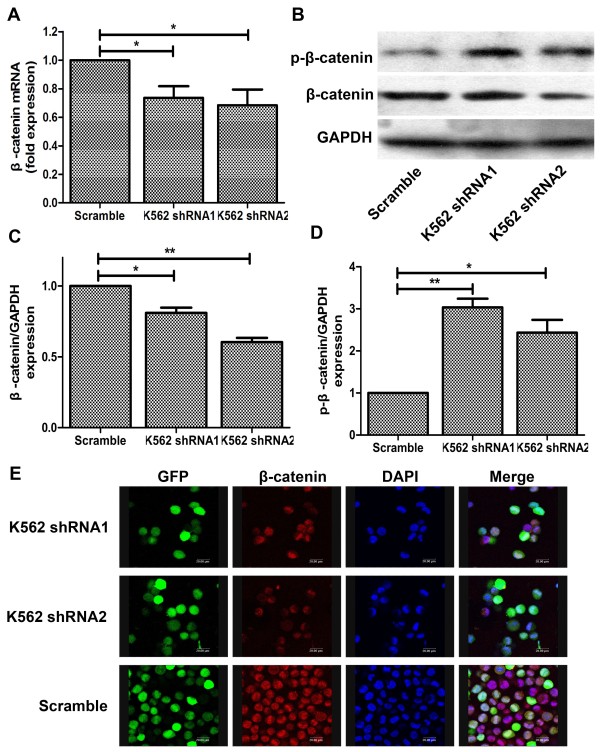
**CD44 suppression inhibited nuclear translocation of β-catenin. (A)** Decreased β-catenin mRNA expression after CD44shRNA transfection. **(B)** The protein expressions of β-catenin and p-β-catenin. **(C/D)** The relative protein expression of β-catenin and p-β-catenin compared to GAPDH. *, P < 0.05, **, P < 0.01, compared with the control. **(E)** Confocal laser microscope showing the decreased nuclear translocation of β-catenin after treatment with CD44shRNA. The green panel is GFP; the red panel isβ-catenin and the blue panel is cell nuclear.

Β-catenin is the central effector molecule of the canonical Wnt signaling pathway, whose deregulation occurs in various malignancies including myeloid leukaemias. So we next sought to investigate whether β-catenin participated in CD44-mediated tumor cell proliferation. A decreased mRNA synthesis was observed when CD44 expressions in K562 cells were silenced (Figure 5A). But the results of western blotting showed the expressions of β-catenin in CD44 silencing K562 cells marginally decreased, especially in CD44 shRNA1 transfecting K562 cells which β-catenin expression almost did not decrease (Figure 5B). We all know that the stability destruction of cytoplasmic β-catenin plays a key role in the signaling output of the canonical Wnt cascade. So we examined the phosphorylation level of β-catenin. As our expectation, CD44 shRNA treatment to K562 cells caused distinguished increased expression of phosphorylated β-catenin (Figure 5B). Β-catenin phosphorylation creates a binding site for the E3 ubiquitin ligase, leading to β-catenin ubiquitination and degradation. Then the nuclear accumulation of β-catenin was determined by immunofluorescence test. The results showed that the nuclear accumulation of β-catenin in CD44 silencing K562 cells dramatically decreased compare to the parental K562 cells, and this outcome was in accord with the phenomenon that the whole protein expression of β-catenin significantly decreased (Figure 5C). These data suggest that β-catenin participated in CD44 mediated cell proliferation mainly though the augmented phosphorylation level of β-catenin decreased its nuclear accumulation.

### Decreased nuclear accumulation of β-catenin caused by CD44 down-regulation inhibits colony-formation *in vitro*

**Figure 6:**
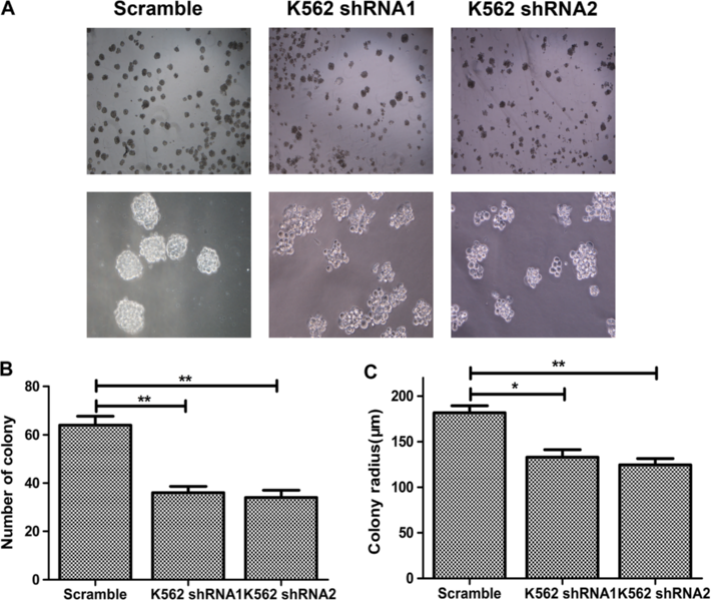
**Effect of decreased expression of CD44 on cell proliferation *in vitro*.** Colony-forming potential was analyzed by semisolid methylcellulose medium. **(A)** Representative photographs of colony formation for K562 cells transfected with CD44shRNA or control vector, respectively. Upper panel (40×) showed the number of colonies, and lower panel (100×) showed the size of colonies. **(B)** Colony numbers for different K562 cell lines of each sight under microscope were shown. **(C)** Colony radius under microscope was also shown. *, P < 0.05, **, P < 0.01, compared with the control.

To investigate whether CD44-mediated decreased β-catenin expression affected the clonal proliferation ability of K562 cells, we compared the *in vitro* growth characteristics by means of colony-formation assays. The previous results showed that transfectants of CD44 shRNA had decreased potential of proliferation, of which the β-catenin protein was accumulated in cytoplasm. In colony-forming assay, CD44 silencing K562 cells formed less colonies than the parental cells (Figure 6A and B). And also, the sizes of colonies formed by CD44 knockdown K562 cells were smaller than the control (Figure 6A and C). These data suggested that the inhibition of CD44 impaired the growth potential of tumor cells compared to wild type or control.

### CD44 down-regulation inhibits the oncogenic potential of K562 cells *in vivo*

**Figure 7:**
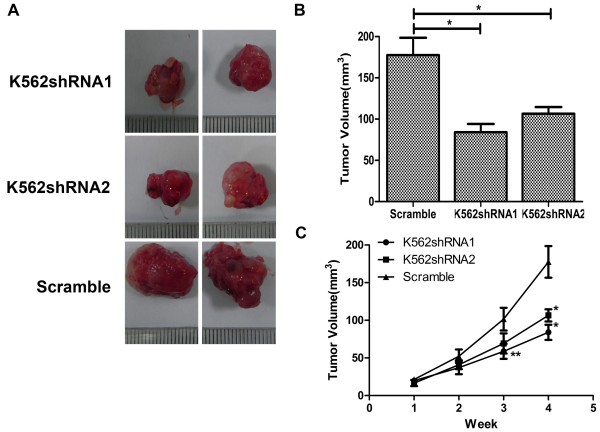
**Down-regulated expression of CD44 impacted cell proliferation *in vivo*. (A)** Representative pictures of tumor taken at the time of killing were shown. **(B)** Volume of subcutaneously formed tumors. Tumor volume was calculated asπLW^2^/6 for 30 days. **(C)** subcutaneous tumor outgrowth of CD44 shRNA and scramble vector-expressing K562 cells. The experiment was carried out blinded and repeated twice with similar results. Data shown were averages from five mice in each group and were presented as Mean ± SD. *, P < 0.05 by Student’s *t*-test.

We next examined whether down-regulation of CD44 altered the tumorigenic capacity of K562 cells *in vivo*. When cells were inoculated subcutaneously, tumors formed in the CD44shRNA group were consistently smaller in size than those in the control group. At the end, the total mass of the tumors harvested from the scramble group was nearly threefold more than that of the CD44shRNA group (P < 0.05) (Figure 7A-B). In addition to a small in tumor size, CD44 knockdown K562 cells showed a significant reduction in tumor outgrowth after subcutaneous injection when compared with control K562 cells (Figure 7C). Furthermore, the scramble transfectants appeared to be more aggressive, invading into many abdominal organs, such as spleen, liver and kidney than K562 cells with CD44shRNA (data was not shown). Collectively, our present findings strongly suggest that the down-regulation of CD44 significantly decreases the tumorigenic capacity of K562 cells *in vivo*.

## Discussion

Chronic myeloid leukemia (CML) is a clonal myeloproliferative disorder that is characterized by a t (9; 22) translocation, which results in the expression of BCR-ABL fusion oncoproteins that are unique to the leukemic cells. This fusion oncoprotein is responsible for the increased activation of several downstream signaling pathways, which affect malignant cells’ behaviors and are necessary for oncogenesis and potential immunogenic [17,18]. The BCR-ABL tyrosine kinase inhibitor imatinib is effective as a single agent for the treatment of patients in all stages of CML, with the most encouraging results seen in patients in chronic phase (CP) disease. Hematologic and cytogenetic responses to imatinib for the treatment of CP CML have permitted imatinib to be registered as first-line treatment for newly diagnosed CML [19,20]. Despite the success of imatinib and other tyrosine kinase inhibitors (TKIs), CML remains largely incurable, and this is likely due to the treatment resistance of leukemic stem cells, which are responsible for rapid disease relapse after the discontinuation of therapy [21,22]. How to treat CML patients who are resistant to BCR-ABL tyrosine kinase inhibitors is an important and urgent issue for clinical hematology. So making further efforts to understand the molecular signals in CML is indispensable.

A number of studies have aimed at identifying the specific molecules expressed in CML stem cells that correlate with oncogenic behaviors. Among such candidates there is a major cell surface receptor CD44, which is a multifunctional transmembrane glycoprotein expressed in many cells and tissues [23]. CD44 is often expressed as a variety of variant isoforms generated by an alternative splicing mechanism and the expression of certain cell surface receptor CD44 variant (CD44v) isoforms is known to be associated with many physiological and pathological processes [24]. So we detected the expressions of CD44 in leukemic patients and the results showed that the expressions of CD44 in leukemic patients were higher than that in normal control. We also examined the CD44 level K562 chronic myeloid leukemia cells compared with the healthy control and the results were the same with that in patients’ samples. Liqing *et al.* also found that CD44 was a key regulator of AML LSCs to maintain their stem cell properties and may provide a therapeutic strategy to eliminate quiescent AML LSCs [25].

A growing body of literatures implicate that many signaling pathways including Wnt, Hedgehog, Notch and Bmi which regulate normal stem cell developmentare, are also classically associated with cancers. One particular interesting pathway that has also been shown to regulate both self-renewal of stem cell and oncogenesis in different organs is the Wnt/β-catenin signaling pathway [26-29]. And we detected the expression of β-catenin in K562 after treatment with CD44shRNA. The results showed that inhibition of CD44 induced slightly decreased β-catenin level but dramatically increased the expression of p-β-catenin.

The relation between β-catenin and CD44 has been studied in many solid tumors such as breast cancer, prostate cancer and colon carcinoma. Sarkar *et al.* found the prograstrin could up-regulate the expressions of β-catenin and CD44, and subsequently increase the proliferation *in vivo*[30]. From that study a relatively good correlation between CD44 and β-catenin expression pattern could be seen. The study of Han *et al.* suggested that siRNA-mediated down-regulation of β-catenin elevated the E-cadherin expression but reduced the CD44 expressions, which inhibited the invasion and migration of colon cancer cells [31]. Meanwhile, Wielenga *et al.* found the expressions of CD44 family were overexpressed in the colorectal adenoma carcinoma, which may be regulated by β-catenin/Tcf-4 signaling pathway [32]. But in leukemia genesis the relationship between CD44 and β-catenin was little studied. A report from Bjorklund *et al.* suggested that CD44 played an important role in cell adhesion and resistance to lenalidomide in multiple myeloma, which could be mediated by β-catenin [33]. In our study, we found in the CD44 silencing K562 cells the expressions of β-catenin were down-regulated. These different results may be form different cell functions in different cell lines. K562 cell lines are BCR-ABL positive cells, which constitutive express BCR-ABL fusion protein. And β-catenin and CD44 may play different functions in these cells. Krause *et al.* found that CD44 was indispensable for induction of leukemia by BCR-ABL and was specifically required for leukemia stem cell that initiated CML [9]. And a study of Hu *et al.* suggested that β-catenin played an essential role on survival and drug-resistance of leukemia stem cell in mice with BCR-ABL-induced chronic myeloid leukemia [6]. Meanwhile, accumulated evidence showed that there is a deregulation and cross talk among Wnt and other signaling pathway such as Notch in chronic myeloid leukemia [34]. So, CD44 may have a cross talk with β-catenin through BCR-ABL and there may be a regulated loop between CD44 and β-catenin.

To ensure the effects of CD44 on proliferation, we down-regulated the CD44 level by shRNA and found that the proliferation of K562 cells significantly decreased compared with that of the parental cells. The inhibition of proliferation was major from the CD44 down-regulation induced a G_0_/G_1_ arrest in cell cycle of K562 cells. These data indicate that the effects of CD44 on cell proliferation are partially contributable to the G_0_/G_1_ arrest of cell cycle in K562 cells. Cell cycle progression through G_1_ to S and the G_2_ to M transitions are major checkpoints in the control of cells’ proliferation. Cyclins, cyclin-dependent kianses(CDK), and cyclin-dependent kinase inhibitors (CDKIs) play important roles in the above processes[35-37]. Among these regulators, Cyclin D1 is indispensable in regulating the G_1_ checkpoint. The expression of Cyclin D1 was usually over-expressed in some kinds of tumor cells such as the invasive breast cancer and ductal carcinoma [38]. On the other hand, these kinase activities of Cyclin/CDK are negatively mediated by CDKIs families such p21 [39]. But the relationship between Cyclin D1 and p21 was not simply negative. Ashrafi *et al.* found that in the breast cancer of wistar albino female rats, the expressions of Cyclin D1 and p21 were all highky up-regulated [38]. In our study, we found the down-regulation of CD44 induced the decreased Cyclin D1 expression and increased p21 expression. Sengupta *et al.* found that β-catenin, CyclinD1, HoxA10 and p21 play important role in the signaling network for the apparently diverse but mutually interconnected self-renewal-associated genetic programs of CML cells and this finding was consistent with our results [34].

## Conclusion

Taken together, in our study we investigate the direct regulating correlation between CD44 and β-catenin in K562 cells. These data demonstrate that down-regulation of CD44 greatly decreases the proliferation by a G_0_/G_1_ arrest of cell cycle in K562 cells. And in the process of CD44-mediated cell proliferation, β-catenin is a target of CD44 to regulate the expression of p21 and cyclin D1. Our findings provide a theoretical basis that simultaneously targeting to CD44 and β-catenin may be novel therapeutic strategies for treating CML.

## Methods

### Cell culture and material

We collected born marrow samples of patients in Hospital of Blood Diseases. We chose 4 acute myeloid leukaemia (AML) patients, 4 chronic myeloid leukemia (CML) patients, 4 acute lymphoblastic leukemia (ALL) patients, 3 myeloproliferative neoplasm (MPN) patients, 3 polycythemia vera (PV) patients, 2 essential thrombocythemia (ET) patients and 2 healthy volunteers. All patients are newly diagnosed. In all CML patients, three patients were in chronic phase and one patient was in accelerated phase. And all AML patients were diagnosed as AML-M3. Inclusion criteria for our study were based on the European Leukemia Net (ELN) criteria. Clinical evaluation of patients was performed with physical examination and laboratory monitoring. All the patient samples were treated in accordance with the Helsinki Declaration. Before the start of treatment, each patient gave written informed consent.

K562 cells were grown in RPMI 1640 (Gibco-BRL Life Technologies, Inc. Burlington, ON, Canada) supplemented with 10% fetal bovine serum (HyClone, Logan, UT), 100 U/ml penicillin, and 100 μg/ml streptomycin at 37°C in a humid atmosphere with 5% CO_2_.

### RNA interference studies, real-time quantitative PCR and Western blotting

These analyses were performed as described previously [40]. Trizol kit (Invitrogen, Grand Island, NY) was used to isolate total RNA and the concentration of total RNA was measured by spectrophotometer. Real-time quantitative PCR (see Table 1 for primers) was performed with ABI 7500 system Instrument with SYBR Green PCR kit (Takara, Japan).

**Table 1:** Prime sequences

**Prime set**	**Forward**	**Reverse**
GAPDH	5′-GAAGGTGAAGGTCGGAGTC-3′	5′-GAAGATGGTGATGGGATTTC-3′
CD44	5′-ACCCCAACTCCATCTGTG C-3′	5′-TTCTGGACATAGCGGGTG-3′
p21	5′-CCCGTGAGCGATGGAACTTG-3′	5′-TGCCTCCTCCCAACTCATC-3′
Cyclin D1	5′-GCGGAGGAGAACAAACAGAT-3′	5′-TGAGGCGGTAGTAGGACAGG-3′
β-catenin	5′-CATCATCGTGAGGGCTTACTG-3′	5′-TGAAGGCAGTCTGTCGTAATAG-3′

The total proteins of K562 cells were extracted and separated by SDS-PAGE. And the protein bands were detected using ECL Western blotting detection kit (GE healthcare, UK). For western blotting analysis, we purchased antibodies GAPDH from Santa Cruz Biotechnology (Santa Cruz, CA); Cyclin D1, p21, Bcl-xl from Cell Signaling Technology (Cell Signaling Technology, USA); CD44 antibody from R&D systems.

### Cell proliferation assay

To assess the proliferation state of K562 cells after various treatments, MTT proliferation assay was performed according to the manufacturer’s instructions. Briefly, K562 cells were seed in 96-well plates at a density of 4 × 10^4^ cells/ml for 1–3 days. A volume of 20 μl MTT labeling reagent (5 mg/ml) was added every day to each well and the plates were incubated at 37°C for 4 h. The resulting formazon crystals were solubilized by adding 100 ml of solubilization buffer (10% SDS in 0.01 m HCl) per well and the plates were incubated at 37°C overnight. The absorbance of the formazon measured at 575 nm was used to account for the proliferation state of cells. Trypan blue cell exclusion was also used to assess the cell viability and the cell number.

### Flow cytometry

K562 cell surface staining, cell cycle, and apoptosis analysis was performed by flow cytometry with a BD LSRII flow cytometer.

For cell surface staining analysis, K562 Cells were pre-incubated with CD44 primary antibody for 1 h at 4°C. After three washes, the K562 cells were incubated with cy5-conjugated Goat Anti-Mouse IgG secondary antibody at room temperature. After analysis, the experiment was analyzed with software of Flowjo 7.6.

For cell cycle analysis, K562 cells in exponential growth phase were permeated with 75% ethanol for overnight at 4°C and stained with Propidium Iodide (PI) in the presence of 5 μg/ml RNase (Sigma) for 10 min. And cell cycle distribution (G0-G1, S and G2-M) was analyzed with DNA cell cycle analysis software (ModFit, Becton Dickinson).

Apoptosis was measured with a commercial kit (Tianjin Sungene Biotech, China) as recommended by the manufacturer. Approximately, 10^5^ K562 cells were stained for 15 minutes with Annexin V- allophycocyanin (APC) and PI at room temperature in the dark. After analysis, the apoptotic K562 cells were analyzed with software of Flowjo 7.6.

### Methylcellulose colony formation assays

K562 cells were harvested and washed three times with IMDM. The cell suspension (2 × 10^3^ cells/ml) were cultured in semisolid methylcellulose medium (H4100, Stem Cell Technologies, Canada) supplemented with 10% FBS. Cells were incubated at 37°C with 5% CO_2_, and the total number of colonies was counted after 10 days by use of an inverted microscope.

### Immunofluorescence assay

K562 cells were fixed with 4% paraformaldehyde for 30 min. K562 Cells were washed with ice-cold PBS, blocked with 0.5% BSA in PBS for 30 minutes and then pre-incubated with CD44 primary antibody overnight at 4°C. After three washes, the K562 cells were incubated with cy3-conjugated AffiniPure Goat Anti-Mouse IgG secondary antibody at room temperature and stained nuclei with 1 μg/μl DAPI. Then K562 cells were washed twice by PBS and the images were visualized with Bio-Rad 1024 confocal laser microscope.

### Inoculation of nude mice

All animal experiments were performed in compliance with the guidelines of Laboratory Animal Care of National Institutes of Health for the care and use of laboratory animals. CD44 shRNA stable transfectants and its parental K562 cells were tested for their tumorigenic potential *in vivo* using nude mice. Five 4-week-old male BALB/C-nu/nu mice were included in each group. In subcutaneous models, 3 × 10^6^ cells suspended in 0.1 ml PBS were injected into the right flank of each mouse at a single site. Tumor length and width were measured every week after injection. Volume was calculated as πLW^2^/6. All mice were kept in aseptic cages and killed 4 weeks after inoculation by cervical dislocation.

### Statistical analysis

Each experiment was repeated at least three times. All data were summarized and represented as mean ± SD. The difference between means was statistically analyzed using the *t*-test. All statistical analyses were performed using GraphPad Prism software (San Diego, USA). p < 0.05 was considered as statistically significant.

## Abbreviations

CML: Chronic myeloid leukemia; LSC: Leukemia stem cells; Ph: Philadelphia.

## Competing interests

We state here none of our authors has financial or other competing interest that might be construed as influencing the results or interpretation of our study.

## Authors’ contributions

GQC, QHL and TXP provided the experimental design; GQC and LM provided the experiments; GQC, HJZ, JW, YJZ, HX, CJW and HRZ analyzed the interpretation of data; GQC wrote the article and all authors gave final approval of manuscript submitted.
